# Long-term behavior at foraging sites of adult female loggerhead sea turtles (*Caretta caretta*) from three Florida rookeries

**DOI:** 10.1007/s00227-014-2415-9

**Published:** 2014-03-22

**Authors:** Allen M. Foley, Barbara A. Schroeder, Robert Hardy, Sandra L. MacPherson, Mark Nicholas

**Affiliations:** 1 Jacksonville Field Laboratory, Fish and Wildlife Research Institute, Florida Fish and Wildlife Conservation Commission, Jacksonville, FL 32218 USA; 2 National Marine Fisheries Service, Office of Protected Resources, Silver Spring, MD 20910 USA; 3 Fish and Wildlife Research Institute, Florida Fish and Wildlife Conservation Commission, St. Petersburg, FL 33701 USA; 4 US Fish and Wildlife Service (retired), Jacksonville, FL 32256 USA; 5 National Park Service, Gulf Islands National Seashore, Gulf Breeze, FL 32563 USA

## Abstract

We used satellite telemetry to study behavior at foraging sites of 40 adult female loggerhead sea turtles (*Caretta caretta*) from three Florida (USA) rookeries. Foraging sites were located in four countries (USA, Mexico, the Bahamas, and Cuba). We were able to determine home range for 32 of the loggerheads. One turtle moved through several temporary residence areas, but the rest had a primary residence area in which they spent all or most of their time (usually >11 months per year). Twenty-four had a primary residence area that was <500 km^2^ (mean = 191). Seven had a primary residence area that was ≥500 km^2^ (range = 573–1,907). Primary residence areas were mostly restricted to depths <100 m. Loggerheads appeared to favor areas with larger-grained sediment (gravel and rock) over areas with smaller-grained sediment (mud). Short-term departures from primary residence areas were either looping excursions, typically involving 1–2 weeks of continuous travel, or movement to a secondary residence area where turtles spent 25–45 days before returning to their primary residence area. Ten turtles had a secondary residence area, and six used it as an overwintering site. For those six turtles, the primary residence area was in shallow water (<17 m) in the northern half of the Gulf of Mexico (GOM), and overwintering sites were farther offshore or farther south. We documented long winter dive times (>4 h) for the first time in the GOM. Characterizing behaviors at foraging sites helps inform and assess loggerhead recovery efforts.

## Introduction

After nesting, female sea turtles migrate back to foraging sites. For cheloniids, this usually involves traveling a distance of at least a few hundred km. The upper limit on migration distance for these adult sea turtles is almost 3,000 km, a trait they share with other aquatic taxa (Hays and Scott 2013). There is plasticity in the use of oceanic versus coastal foraging sites both across and within the cheloniid species. In loggerheads, some postnesting females migrate to oceanic feeding areas (Hatase et al. 2002; Hawkes et al. 2006; Rees et al. 2010), but most travel to neritic foraging sites (Luschi et al. 2006; Broderick et al. 2007; Hawkes et al. 2007; Zbinden et al. 2008; Girard et al. 2009; Marcovaldi et al. 2010; Rees et al. 2010; Hart et al. 2012). Those foraging in neritic zones usually remain in one area all year (Marcovaldi et al. 2010) or occupy one in the summer and another in the winter (Broderick et al. 2007; Hawkes et al. 2007; Zbinden et al. 2008). These residence areas are typically a few hundred square km in size although some may be as large as 1,000–2,000 km^2^ (Broderick et al. 2007; Zbinden et al. 2008; Hawkes et al. 2011; Hart et al. 2012). The foraging sites of adult male loggerheads are largely the same as those of adult females, although there may be a tendency for males to choose sites that are closer to breeding or nesting areas (Arendt et al. 2012, Schofield et al. 2013). Describing the location and extent of foraging areas for all adult loggerheads is essential when designating conservation areas.

The loggerheads in the Northwest Atlantic Ocean have been recognized as a distinct population segment (DPS) and are listed as threatened under the US Endangered Species Act (Department of the Interior and Department of Commerce 2011). This DPS includes one of only two major loggerhead nesting assemblages in the world (National Marine Fisheries Service and US Fish and Wildlife Service 2008), and about 90 % of this nesting occurs in Florida (Ehrhart et al. 2003). Promoting recovery of the loggerhead populations that nest in Florida is vital to the Northwest Atlantic Ocean DPS and to the species as a whole (National Marine Fisheries Service and US Fish and Wildlife Service 2008). Reducing threats to adult loggerheads is important to these recovery efforts because the survival rate of adults has a particularly strong effect on population recovery (Crouse et al. 1987; Heppell 1998).

Incidental take in commercial fisheries is the greatest threat to adult loggerheads in neritic areas of the Northwest Atlantic (National Marine Fisheries Service and US Fish and Wildlife Service 2008). An essential step in any attempt to minimize this mortality is to identify the foraging areas of adult loggerheads and their behavior at these sites. This is needed to determine where, when, and how foraging loggerheads and fisheries overlap. Other anthropogenic threats such as vessel strikes, oil and gas activities, power-generating activities, and military activities might also be mitigated if the spatial and temporal distributions of adult loggerheads in foraging areas, in addition to their behaviors, were well documented.

The overall objective of our study was to document, using satellite telemetry, the postnesting migrations and subsequent behavior in foraging areas of adult female loggerheads from three Florida rookeries. We have already described the postnesting migrations of these turtles (Foley et al. 2013). Our goals in the present study were to determine the locations of foraging sites, to characterize them, and to document the movements and other behaviors of the loggerheads at these sites.

## Materials and methods

We intercepted loggerheads from three Florida rookeries (northwestern, central western, and central eastern; see Shamblin et al. 2011) after they had nested and confined them in a bottomless wooden box with 8-cm holes along the sides for ventilation. We affixed a platform transmitter terminal (PTT) to the flattest part of the first three vertebral scutes of each turtle according to the methodology of Balazs et al. (1996). A description of the PTTs and their duty cycles are given in Foley et al. (2013). Ten of the PTTs were equipped with a pressure sensor that collected dive data every 10 s. These transmitters documented two diving parameters that were used in the present study: time at depth (min m^−1^) and dive duration (min). Time at depth was the proportion of time spent within defined depth bins. Depth bin limits were set at 1, 3, and 5 m, then at 5-m intervals to 35, then at 50, 60, and 75 m, then at 25-m intervals to 150 m, and then at >150 m. The duration of each dive was also assigned to defined time bins. Time bin limits were set at 2, 5, 10, 20, 30, 45, and 60 min, then at 30-min intervals to 240 min, and then at >240 min.

The process used to determine positions from transmissions between the PTTs and an Argos receiver and the potential error associated with these positions are described by Foley et al. (2013). Argos data were downloaded and managed using the Satellite Tracking and Analysis Tool (Coyne and Godley 2005). To exclude implausible locations, we evaluated the Argos positions using a standardized set of user-defined movement rules that were implemented by the Douglas Argos Filter Algorithm (DAF) written for PC SAS (Douglas 2006). The beginning of the period associated with long-term foraging (endpoint of the postnesting migration) was identified by a reduction in travel rate to less than 20 km day^−1^, a cessation of net movement away from the nesting beach, and an end to primarily unidirectional orientation. Locations during this time were determined with positions from the DAF minimum redundant distance output, which produced a subset of Argos data that passed tests for plausibility based on spatial redundancy (within 5 km) of near-consecutive positions. These positions were then reduced to the best per day by ranking them individually with respect to location class, residual error of the frequency calculation, number of messages received, and transmitter oscillator frequency drift (Douglas 2006; Collecte Localisation Satellites 2008).

We used three home-range estimators to calculate the size of loggerhead home ranges in foraging areas from the filtered Argos positions. These were minimum convex polygons (MCPs), MCPs that were refined using the *α*-Hull technique (Burgman and Fox 2003, using *α* value of 3 as recommended by Hawkes et al. 2011), and fixed-kernel density (FKD) analyses using least-squares cross-validation smoothing (Gitzen and Millspaugh 2003). Home-range size was quantified using the adehabitat package (Calenge 2006) within R (R Development Core Team 2011) and Home Range Tools (Rodgers et al. 2007) within ArcGIS 9.3 (Environmental Systems Research Institute 2009). Following a recommendation of Laver and Kelly (2008), we graphed the calculated size of the home range of each loggerhead with an increasing number of tracking days to determine the time at which further increases in the calculated size of the home range were minimal. We also expected that at least 30 positions might be necessary to allow accurate calculation of the size of the home range based on computer simulations performed by Seaman et al. (1999).

We obtained bathymetric summaries of foraging areas using two regional data sets. The 3-arc-second-resolution US coastal relief model (CRM, NOAA National Geophysical Data Center 2009) was used for loggerheads with foraging sites that were entirely within the US coastal zone. For loggerhead foraging sites that were beyond the extent of the CRM, we used bathymetry values from the global (1-arc-minute resolution) ETOPO1 data set (Amante and Eakins 2009).

We characterized the seafloor at loggerhead foraging sites on the west Florida continental shelf (WFS) using the Gulf of Mexico (GOM) Data Atlas (Jenkins 2011). This data set described the dominant benthic sediment type at a resolution of a 0.02° (4.4 km^2^) grid. The sediment types (by particle size) were mud (<63 μm), sand (≥63 μm and ≤2 mm), gravel (>2 mm and <256 mm), and rock (≥256 mm). We conducted a chi-square test for independence to compare the frequency of sediment types on the entire WFS to that in the home ranges of the loggerheads on the WFS using the software SigmaStat for Windows version 3.10 (with an alpha level of 0.05).

## Results

**Fig. 1:**
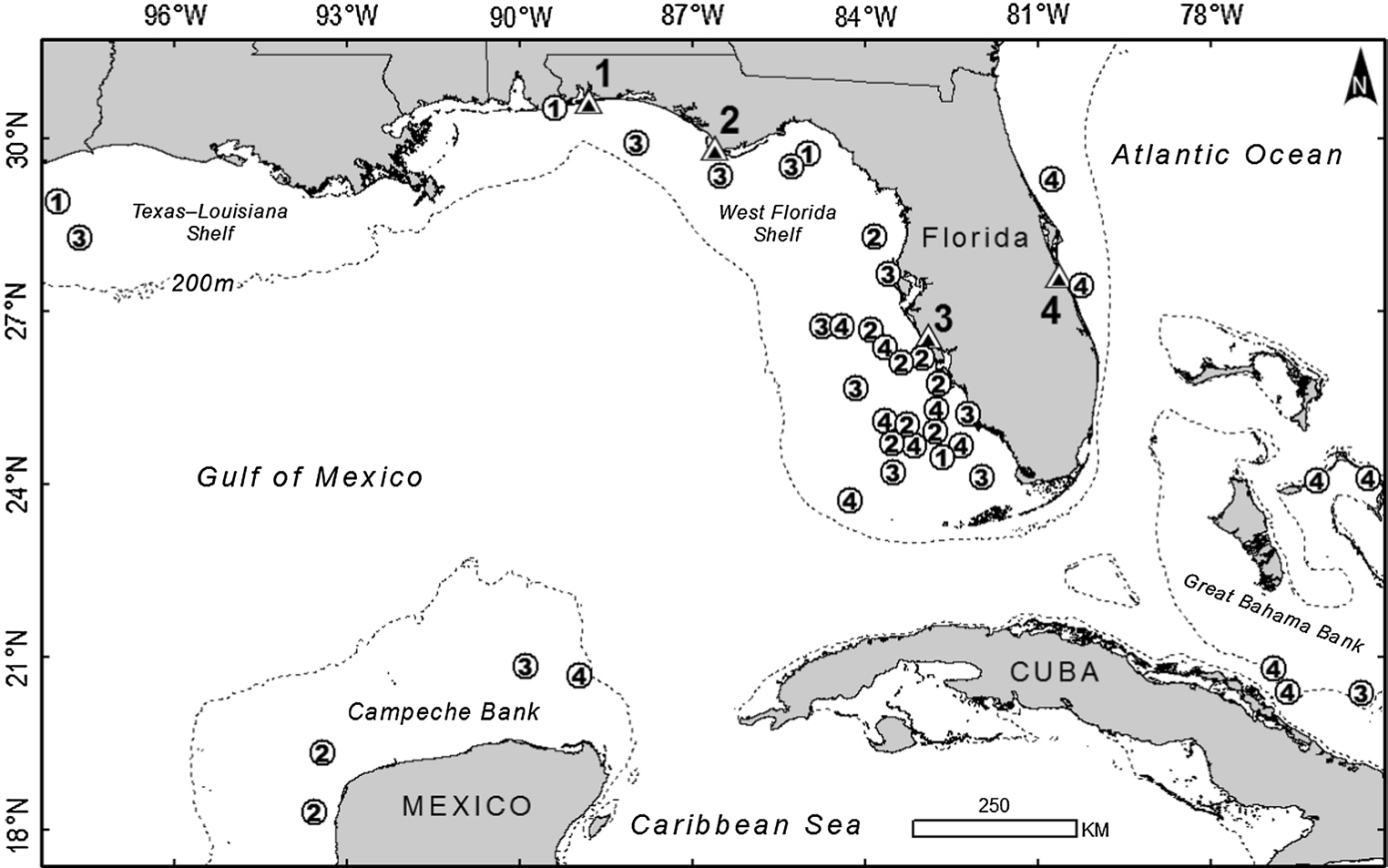
Geographic centers of residence areas for 40 loggerheads outfitted with a platform transmitter terminal during 1998–2001 after nesting in Florida. The location of each nesting beach is denoted by a *triangle* and identified by a number. The location of each residence area is denoted by a *circle* with a number that corresponds to the nesting beach of that loggerhead. Some residence areas on the West Florida Shelf had more overlap than indicated (some *circles* were moved slightly to make them visible). The *dotted line* shows the shelf break (at 200 m)

We affixed PTTs to 42 loggerheads during 1998–2001. Fourteen turtles were from the northwestern rookery, 13 were from the central western rookery, and 15 were from the central eastern rookery (see Fig. 1 in Foley et al. 2013 for nesting beach locations and rookery delineations). Forty of the loggerheads completed their postnesting migration and exhibited residence behavior at a presumed foraging site before their tracking period ended. These foraging sites were on the continental shelf of the states of Florida, Alabama, and Texas, USA; Mexico; the Bahamas; and Cuba (Fig. 1), and were on average 501 km (shortest distance by water) from each turtle’s nesting beach (SE = 46.8, range = 27–1,143).

**Fig. 2:**
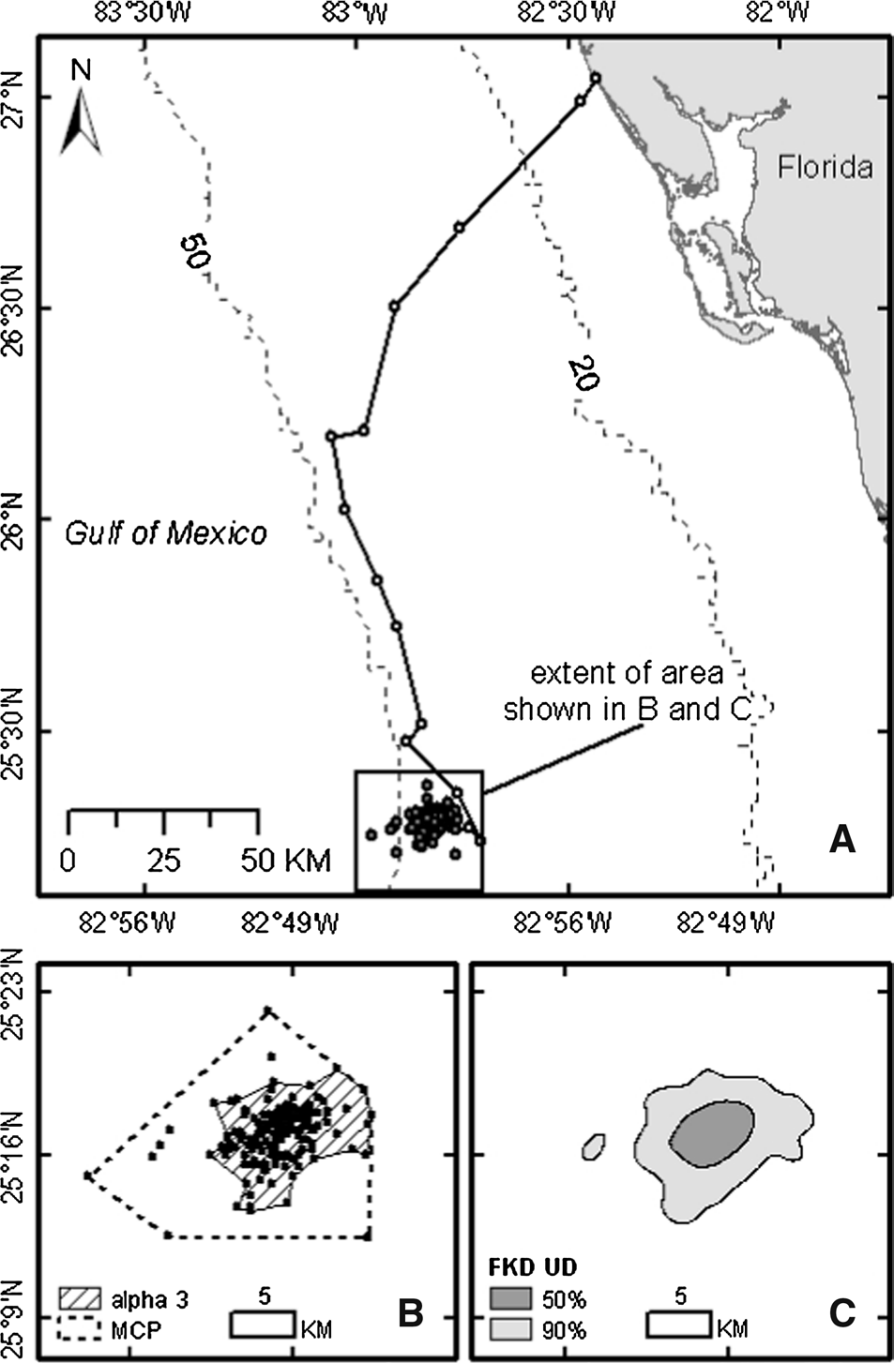
Loggerhead primary residence area as indicated by filtered positions and as described by a minimum convex polygon (MCP), an *α*-Hull (where *α* = 3), and a FKD of 90 and 50 %. This loggerhead was outfitted with a platform transmitter terminal on July 28, 2000, after nesting in western central Florida. **a** Spatial representation of the postnesting migration (*open points*, 14 days) and subsequent primary residence area (*closed points*, 237 days). **b** Extent of primary residence area as determined by an MCP (241 km^2^) and an *α*-Hull analysis (77 km^2^). **c** Extent of primary residence area as represented by an FKD of 90 % (99 km^2^) and 50 % (22 km^2^)

**Fig. 3:**
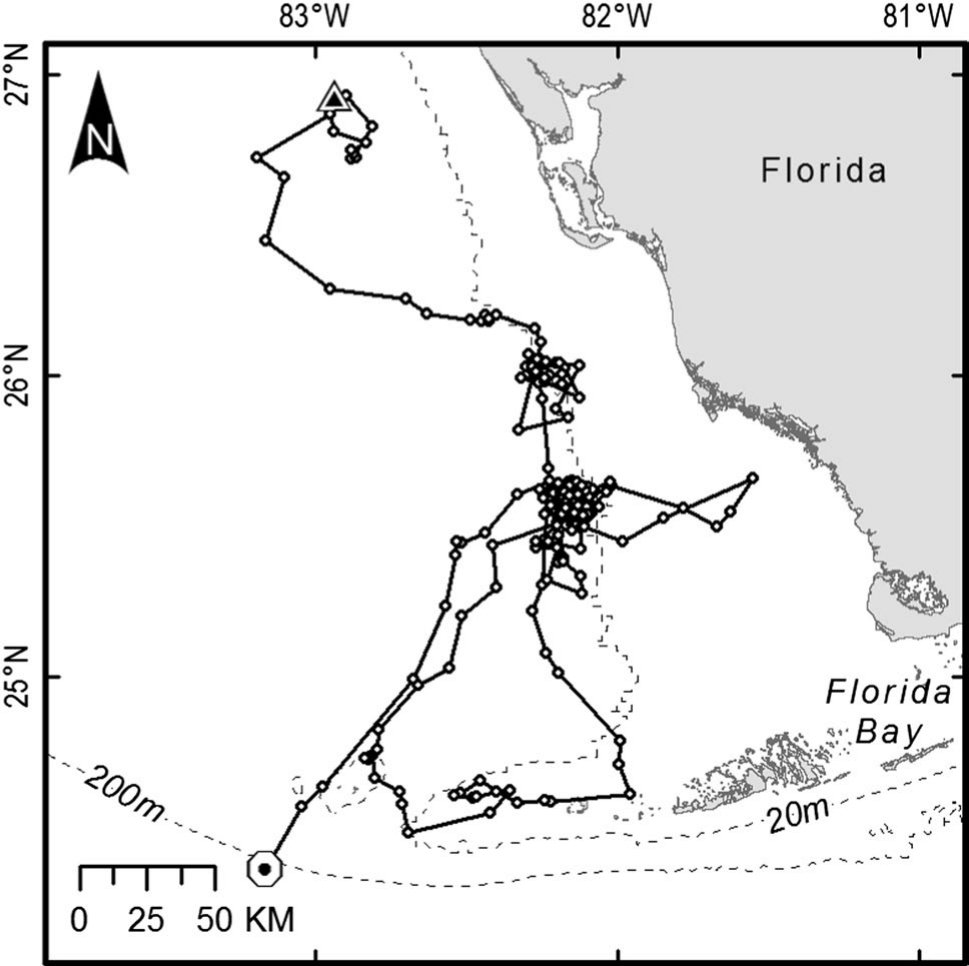
The movements of a loggerhead on the west Florida continental shelf (WFS) after completing its postnesting migration. This turtle was outfitted with a platform transmitter terminal on July 14, 2001, after nesting in northwestern Florida. The *triangle* denotes the point at which the track begins (end of postnesting migration), and the *circle* denotes the point at which the tracking period ended. This loggerhead had several areas of temporary residence (1–3 months), while otherwise ranging over 28,000 km^2^ of the southern portion of the WFS over a period of 214 days

We did not collect an adequate number (at least 30) of filtered positions to delineate the home range at foraging sites for eight of the loggerheads. For the remaining 32 loggerheads, we collected a mean of 197 filtered positions (SE = 19.2, range = 32–404) at foraging sites over a period of at least 100 days (mean = 396 days, SE = 26.9, range = 107–706). The mean numbers of filtered positions by location class for these turtles are given in Table 1. For 31 of these loggerheads, the initial area of residence after completing the postnesting migration was where each turtle spent most (usually >11 months per year) or all of its time. We refer to this area as the *primary residence area*. Examples of a primary residence area as described by an MCP, an *α*-Hull (where *α* = 3), and a FKD of 90 and 50 % are shown in Fig. 2. One loggerhead that was tracked for 214 days after completing its postnesting migration, and for which there were 153 filtered positions during this time, did not have a primary residence area. This turtle spent about 4 months in several discrete residence areas on the WFS, interspersed with about 3 months of traveling through, and then eventually beyond the waters of the continental shelf (Fig. 3).

**Table 1:** Mean number of filtered positions by location class used to delineate the primary residence areas of 32 loggerheads that were outfitted with a platform transmitter terminal after nesting in Florida during 1998–2001

Location class	Mean number of filtered locations
3	9.3 (2.1, 0–47)
2	22.6 (3.8, 0–67)
1	42.0 (6.8, 1–132)
0	32.2 (4.7, 1–86)
A	40.3 (4.1, 4–83)
B	48.9 (6.1, 4–156)
Z	1.7 (0.3, 0–6)
All	198.3 (19.7, 32–404)

The standard error and range are given in parentheses. Accepted positions were initially those from the Douglas Argos Filter minimum-redundant distance output. Those were then reduced to the best position per day by individually ranking them based on location class, residual error on the frequency calculation, number of messages received, and transmitter oscillator frequency drift (Douglas 2006; Collecte Localisation Satellites 2008)

We graphed the calculated size of each primary residence area (not including any looping excursion or secondary residence area, see below) of each of the 31 loggerheads over time and found that after 100 days of tracking, further increases in the calculated size of this area were typically minimal. For these turtles, we found no correlation between the size of the primary residence area (as delineated by the *α*-Hull) and the number of positions used to calculate the area (Pearson product-moment correlation, *r* = 0.160, *P* = 0.390) or the number of days over which the turtle was tracked (Pearson product-moment correlation, *r* = 0.273, *P* = 0.137).

The sizes of the primary residence areas as determined from the three home-range estimators are given in Table 2. The mean depth in the primary residence areas (as delineated by the *α*-Hull) was 29.8 m (SE = 3.9, range = 1.3–80.3), and all but three were restricted to a depth of <100 m. A comparison of the frequencies of the dominant sediment types on the entire WFS to that in the 19 primary residence areas of loggerheads on the WFS (as delineated by the *α*-Hull) is shown in Table 3.

**Table 2:** Mean sizes of the primary residence areas of 31 loggerheads that were outfitted with a platform transmitter terminal during 1998–2001 after nesting in Florida

General location of residence area	Number of turtles	MCP (km^2^)	*α*-Hull (km^2^)	FKD 90 % (km^2^)	FKD 50 % (km^2^)
West Florida Shelf	14	411 (55.9, 119–921)	191 (27.7, 77–435)	251 (53.3, 97–865)	66 (18.5,18–289)
West Florida Shelf	5	1,967 (302.5, 878–2,731)	867 (102.9, 573–1,053)	1,176 (372.9, 525–2,628)	326 (119.7, 134–795)
East Florida Shelf	1	805	319	299	67
East Florida Shelf	1	1,403	745	782	128
Texas–Louisiana Shelf	1	761	242	269	60
Texas–Louisiana Shelf	1	3,959	1,907	1,611	386
Campeche Bank, Mexico	4	432 (103.2, 211–691)	151 (25.0, 87–207)	156 (28.9, 80–215)	30 (5.1, 17–42)
Great Bahama Bank	4	482 (87.7, 261–667)	186 (29.7, 124–261)	200 (62.5, 122–387)	47 (16.9, 29–98)

The standard error and range are given in parenthesis when appropriate. MCP is the minimum convex polygon. For the *α*-Hull analysis, the value of *α* was set at 3. FKD is the fixed-kernel density at 90 and 50 %. Turtles with primary residence areas in each general location were divided between those with an *α*-Hull of <500 km^2^ and those with an *α*-Hull of ≥500 km^2^

**Table 3:** The number and percentage of 0.02° grids (4.4 km^2^) dominated by each of the four benthic sediment types on the entire continental shelf of west Florida (WFS) and within the primary residence areas (as delineated by an *α*-Hull, where *α* = 3) of 19 loggerheads on the WFS

Dominant sediment type	Number of 0.02° grids (%)	
	Entire WFS	Primary residence areas
Mud	4,165 (10.5 %)	24 (1.6 %)
Sand	26,419 (66.8 %)	898 (60.7 %)
Gravel	6,818 (17.2 %)	435 (29.4 %)
Rock	2,152 (5.4 %)	122 (8.2 %)

Sediment data are from Jenkins (2011), who determined which sediment type was predominant within each grid. The frequency of grids dominated by each of the four sediment types on the entire WFS was different from that in the primary residence areas of the loggerheads (chi-square test for independence, *χ* ^2^ = 258.5, *df* = 3, *P* < 0.001)

**Fig. 4:**
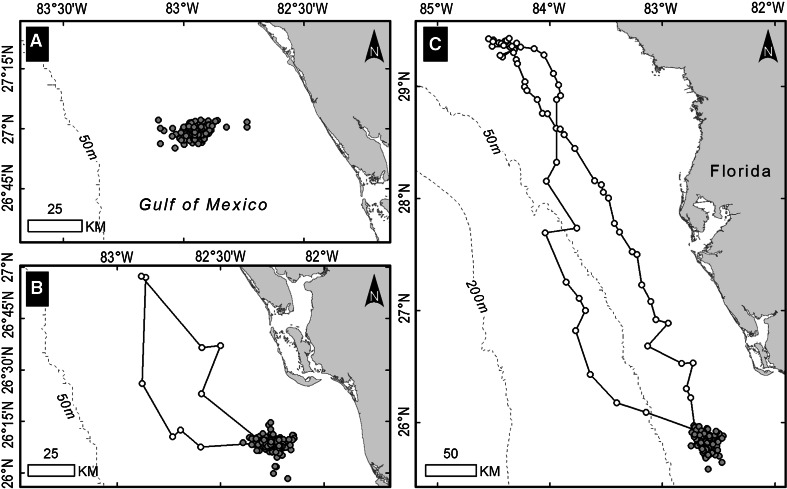
Examples of residence behavior at foraging sites of three loggerheads (turtles **a**–**c**). All three were outfitted with a platform transmitter terminal during August 1999 or August 2000 after nesting in central eastern Florida. Turtle **a** spent the entire tracking period (350 days) within the primary residence area; turtle **b** made a 12-day looping excursion from the primary residence area during a 393-day tracking period; and turtle **c** had a secondary residence area. Turtle **c** spent 211 days in the primary residence area (area to the southeast) and 30 days in the secondary residence area (area to the northwest)

Sixteen of the loggerheads spent the entire tracking period within the primary residence area (see example in Fig. 4a). These 16 turtles were tracked over a mean period of 415 days (SE = 37.2, range = 107–650). The other 15 loggerheads with primary residence areas each made at least one short-term departure from that area. None of these departures involved travel beyond the continental shelf. One type of departure was characterized by a continuous path that led away from the primary residence area and eventually looped back (Fig. 4b). Nine of the loggerheads made this type of *looping excursion*, which usually lasted 1–2 weeks and covered 100–300 km. Looping excursions were made mostly during spring and fall. Seven of the turtles made one looping excursion; two of the turtles made two looping excursions, the second following approximately the path of the first. The other type of departure from the primary residence area led to another area of occupancy that we call the *secondary residence area* (Fig. 4c). Eight of the loggerheads had a secondary residence area, which they usually occupied for 25–45 days. The secondary residence area was typically within 100 km of the primary residence area, but for one loggerhead, it was 400 km away. Two loggerheads moved to their secondary residence areas during the summer, and two moved there during the fall. Only one of these turtles was still being tracked 1 year later, and it moved again to the same secondary residence area again in the fall. The other four loggerheads with secondary residence areas moved to these areas during the winter. For one, this overwintering location was 160 km south of its primary residence area. For the other three, the overwintering site was 30–80 km farther offshore than their primary residence area. Two of the turtles with offshore overwintering sites were tracked over two winters and both moved the same distance farther offshore each winter.

**Fig. 5:**
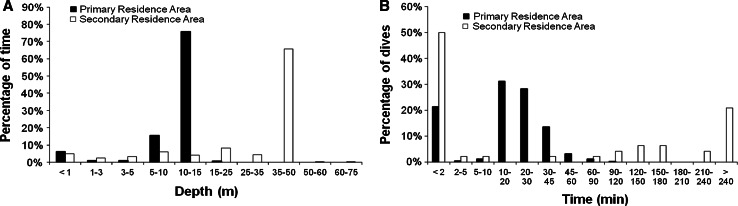
Evidence of a primary and secondary residence area in the dive data of a loggerhead with limited location data (**a**) and frequency of dive durations for this same turtle when in its primary and secondary residence areas (**b**). This turtle was outfitted with a platform transmitter terminal on July 10, 2000, after nesting in northwestern Florida. The residence areas were on the west Florida continental shelf. The dive data indicated that there was a shallower primary residence area and a deeper (20–40 m) secondary residence area that was used only during the winter (Dec–Feb)

Dive-depth and dive-duration data were collected for 10 loggerheads while resident at foraging sites. These data were collected during at least one winter for five of these turtles, all of which had a foraging site on the WFS. One of these loggerheads was known from its tracking data to have a secondary residence area that was farther offshore and was occupied during the winter, and the dive-depth data at that time reflected the deeper water of the overwintering site. The dive-depth data for two other loggerheads (for which there was not a sufficient number of filtered positions over a long enough period of time to allow us to delineate their home range) suggested that they also had an overwintering site that was farther offshore (see example of one in Fig. 5a). The dive-duration data for four of these five loggerheads (three that moved to an overwintering site that was farther offshore than their primary residence area and one that remained in its primary residence area all year) revealed a distinct change in diving behavior during the winter that included dives of unusually long duration (>4 h, see example in Fig. 5b).

A loggerhead that we outfitted with a PTT on August 8, 2000, when it was nesting in central eastern Florida, was intercepted on the same beach when she nested 2 years later on June 4, 2002. The old PTT was removed, and a new one attached using the same methodology as before. We tracked this loggerhead for 594 days between nesting seasons in 2000 and 2002, and for 386 days after the 2002 nesting season. She remained in a primary residence area during both tracking periods and made no departures except to migrate to and from the nesting beach. After nesting in 2002, this turtle returned to the same primary residence area that she had occupied between the 2000 and 2002 nesting seasons. Approximately 91 % of the primary residence area (as delineated by the *α*-Hull) occupied between the 2000 and 2002 nesting seasons was inside the primary residence area occupied after the 2002 nesting season. Furthermore, the mean center of the filtered positions during residency after the first postnesting migration (*N* = 404) was only 241 m from the mean center of filtered positions during residency after the next postnesting migration 2 years later (*N* = 128).

## Discussion

The locations of foraging sites for most of the adult female loggerheads in the present study were similar to those observed in other studies of loggerheads that nested in Florida (Girard et al. 2009; Ceriani et al. 2012; Hart et al. 2012). Two of our turtles (one that nested in central western Florida and one that nested in northwestern Florida), however, had foraging sites on the continental shelf of the northwestern GOM, southeast of Galveston, Texas. Adult-size loggerheads have been documented in this area (Renaud and Carpenter 1994; Hickerson 2000), but their nesting sites were not known.

None of the 15 loggerheads from the central eastern rookery in the present study had foraging sites in the Atlantic north of Florida. In contrast, all other studies of loggerhead postnesting movements from that rookery found that 20–35 % of the turtles had foraging sites north of Florida. These included studies conducted from 1972 to 1978 (Meylan et al. 1983), from 1988 to 1992 (Dodd and Byles 2003), and from 2008 to 2010 (Ceriani et al. 2012). The results of our study may simply signify that fewer loggerheads that nested in central eastern Florida during the time of our work (1998–2000 for that rookery) had foraging sites north of Florida. Our methodology of intercepting loggerheads from that rookery late in the nesting season (August 9–20), however, could have introduced a bias in our sample that was not present or not as strong in the other studies. Loggerheads from foraging sites in different regions can begin migrations to shared nesting beaches at different times (Limpus 1985). Those with foraging sites in different regions may also begin their postnesting movements at different times in the nesting season (Rees et al. 2010). In the most recent study of postnesting movements of loggerheads from the central eastern Florida rookery (Ceriani et al. 2012), the final nest for four of the six loggerheads that had foraging sites north of Florida had been made by July 19 (with three of them completing their nesting season by July 5, S. Ceriani, pers. comm). The loggerhead in the study by Dodd and Byles (2003) that had a foraging site north of Florida was intercepted relatively early in the nesting season (June 29) and returned to that foraging site shortly thereafter. The other three loggerheads tracked in the study by Dodd and Byles (2003) did not have foraging sites north of Florida, and all were still nesting in late August. At the time we were intercepting nesting females of the central eastern rookery (mid-August), most of the loggerheads with foraging sites north of Florida may already have departed, decreasing the probability that we would encounter representatives of this group.

Some sea turtles, like the green turtles that nest on Ascension Island, have to swim a long distance (>2,000 km) during the postnesting migration because there are no suitable foraging areas near the nesting beach (Mortimer and Carr 1987). Most of the loggerheads that nest in the southeastern United States, however, could find suitable foraging areas just offshore of their nesting beach. Nevertheless, many of the loggerheads in the present study migrated to foraging sites that were closer to the nesting beaches of other loggerhead rookeries, creating a situation in which these turtles were crossing paths during their postnesting migrations. Choosing a foraging site that is relatively far from the nesting beach when apparently suitable sites are closer is a behavioral trait that appears to be common among adult female loggerheads in many parts of their range (Australia, Limpus et al. 1992; Northwest Atlantic, present study and compare Hawkes et al. 2007 and Ceriana et al. 2012; Mediterranean, compare Margaritoulis et al. 2003; Broderick et al. 2007; and Zbinden et al. 2008). Hays et al. (2010) hypothesized that an adult loggerhead might choose a foraging area based on its early life experiences. They revealed similarities between the postnesting migrations of adult loggerheads from a major Greek nesting beach and the likely paths of passively dispersing hatchlings from that beach. Hatchling dispersal off the east coast of Florida primarily follows the northward flowing Florida Current (Witherington et al. 2012). It appears unlikely that these hatchlings would have early life experiences in the GOM, the Bahamas, or Cuba (all requiring southerly dispersal). Other hypotheses such as food abundance predictability and geographic variation in mortality rates have been proposed to explain the distribution of adult cheloniid foraging sites (Van Dam et al. 2008). However, neither seems to account for why (as in the present study) many adult females from one rookery would have foraging sites in relatively close proximity to the nesting beaches of another rookery when adult females from the latter rookery have foraging sites in relatively close proximity to the nesting beaches of the first rookery.

As in other studies (Blumenthal et al. 2006; Hawkes et al. 2006, 2011; Luschi et al. 2006; Broderick et al. 2007; Zbinden et al. 2008; Marcovaldi et al. 2010; Hart et al. 2012), the loggerhead foraging sites in the present study were typically in waters <100 m deep. When assessing the potential importance of neritic areas as foraging habitat for adult loggerheads, considerations could include the amount of area with <100 m of water (assuming that more area could support more turtles), the results of tracking studies, and stranding data (see Schofield et al. 2013). Based on our findings, a further consideration could be benthic sediment type. Loggerheads in the GOM appeared to prefer areas with larger-grained sediment (gravel and rock) to those with smaller-grained sediment (mud). This also appeared to be true for adult female loggerheads along the northern coast of Brazil (Marcovaldi et al. 2010) and could be the case in other areas as well.

There are several features in the northern half of the GOM that could concentrate prey species and attract an unusually dense aggregation of foraging loggerheads. These are the Flower Garden Banks (FGB, on the Texas–Louisiana continental shelf southeast of Galveston), the Florida Middle Grounds (FMG, on the northwestern portion of the WFS), and the Mud Hole Submarine Springs (MHSS, on the southeastern portion of the WFS; see Breland 1980; Fanning et al. 1981; Saleem 2007). These features are at depths typical of loggerhead foraging sites (<100 m), have irregular submarine terrain with sand or sand–gravel substrates, and have limestone escarpments or coral banks of various sizes. None of the loggerheads in the present study were found within the FGB or the FMG, but the foraging sites of two of the loggerheads on the WFS did include part of the MHSS. Loggerheads have been noted in the vicinity of the MHSS, and one of the methods used to locate these springs has been to look in areas where surfacing loggerheads were sighted (Fanning et al. 1981). Numerous oil and gas platforms are found on the continental shelf of the northern GOM from Alabama through Texas, and loggerheads are known to frequent the underwater supports of these structures (Renaud and Carpenter 1994). Two of the loggerheads in the present study had foraging sites on this portion of the shelf, but both were in an area of relatively few platforms (50–150 km southeast of Galveston; see Russell 2005 for the distribution of oil and gas platforms).

We used three techniques to calculate the size of primary residence areas to facilitate comparisons to the sizes of foraging home ranges calculated in other studies. Each should be interpreted differently. MCPs described the maximum extent of a home range based on the location data but probably overestimated the area occupied (Burgman and Fox 2003). The *α*-Hull refinement reduced the MCPs to an area that more likely represented the actual extent of movements (Burgman and Fox 2003). This was the home-range estimator that we preferred. The spatial use distributions derived from FKD analyses demarcated areas with greater densities of filtered positions and were assumed to indicate the most-frequented areas. The FKD of 90 % delineated an area that was similar to that determined by the *α*-Hull, and the FKD of 50 % identified what could be considered a core area of activity. We did not estimate the size of secondary residence areas because we had relatively few filtered positions (usually <20) from these sites.

The size of adult female loggerhead home ranges has been estimated at foraging sites around much of the rim of the Atlantic Ocean, including the Northwest Atlantic (Hawkes et al. 2007, 2011; present study), the GOM (Renaud and Carpenter 1994; Hart et al. 2012; present study), the Southwest Atlantic (Marcovaldi et al. 2010), the Southeast Atlantic (Hawkes et al. 2006), and the Mediterranean Sea (Broderick et al. 2007; Zbinden et al. 2008). Despite the vast spatial separation of these study sites and the variety of study methodologies, the estimated sizes of these home ranges are consistent. The majority of foraging loggerheads have a home range of 300–600 km^2^. As in the present study, however, there has been a consistent minority of adult female loggerheads with an unusually large foraging site, from 1,000 to many thousands of square km.

Despite choosing only the most accurate Argos-derived positions accumulated over a relatively long period, we believe that our estimates of the sizes of foraging loggerhead home ranges (and perhaps most or all of the published estimates) are somewhat crude. This arises primarily from the inescapable error associated with the Argos-derived positions when tracking sea turtles. This characteristic is an artifact of tracking animals that spend most of their time underwater, where the transmitter cannot communicate with satellites (Hays et al. 2001; Vincent et al. 2002). Future estimates of foraging loggerhead home ranges are likely to use GPS-derived positions transmitted through the Argos system. Such positions are more accurate and will likely reveal that loggerhead home ranges are smaller than published studies have estimated (see Witt et al. 2010).

The Argos-derived positions may make it difficult to absolutely define the extent of loggerhead home ranges at foraging sites, but they leave no doubt that these loggerheads usually occupy a discrete foraging site that is often <500 km^2^. Some earlier assessments of loggerhead behavior concluded that adult female loggerheads typically move continuously through a series of coastal foraging areas (Hendrickson 1980; Plotkin 2003). It is now clear, however, that most adult female loggerheads in neritic foraging areas use one or two foraging sites and are faithful to these sites (Limpus and Limpus 2001; Broderick et al. 2007; Hawkes et al. 2011; present study). Nevertheless, some foraging adult female loggerheads do appear to be itinerant. Most are feeding primarily or entirely in oceanic waters (Hatase et al. 2002; Hawkes et al. 2006; Rees et al. 2010), but at least a few have been found to move continuously through neritic foraging areas (Zbinden et al. 2008; Girard et al. 2009; present study).

Adult female loggerheads with two neritic foraging areas had previously been documented as spending at least 3–6 months in each (Broderick et al. 2007; Hawkes et al. 2007; Zbinden et al. 2008). Because of the timing of occupancy, these have been called summer and winter foraging areas. Ten of the loggerheads in the present study occupied two foraging areas, but one area was occupied for only about 1 month per year, and at any time of the year, depending on the turtle. Consequently, we used the terms primary and secondary residence areas rather than summer and winter residence areas.

It has been suggested that environmental conditions in the GOM allow loggerheads to remain at a single foraging site year-round (Hart et al. 2012). However, about 20 % of the loggerheads in the present study with foraging areas in the GOM had a separate overwintering site. It appears that the latitude and depth of the primary residence areas were the main determinants of whether these loggerheads used an overwintering site. Loggerheads with overwintering sites all had a relatively shallow primary residence area (mean depth <17 m) in the northern half of the GOM (five on the WFS and one on the Texas–Louisiana shelf). Phillips (2011) also noted that an adult female loggerhead with a shallow-water foraging site on the WFS moved farther offshore during the winter, but she considered that behavior anomalous.

Adult female loggerheads that reside at lower latitudes tend to remain at a single foraging site all year (Limpus and Limpus 2001; Blumenthal et al. 2006; Hawkes et al. 2006; Marcovaldi et al. 2010), and those at higher latitudes tend to move during the winter to presumed warmer-water areas that are either farther offshore or farther south (Broderick et al. 2007; Hawkes et al. 2007; Zbinden et al. 2008). This dichotomy in behavior has also been observed in loggerheads along the latitudinal gradient of a single study area (Hawkes et al. 2011). Because only the loggerheads with the shallowest primary residence areas in the northernmost part of our study area had overwintering sites, and because the time spent at these sites was unusually short, we believe that this part of our study area is at the boundary (latitudinally, from about 27 to 30°N) between loggerhead populations that remain at one foraging site year-round and those that have summer and winter foraging sites.

After moving to an overwintering site, loggerheads sometimes dramatically alter their diving behavior. In the Northwest Atlantic (Hawkes et al. 2007) and Mediterranean (Broderick et al. 2007), dive durations of adult female loggerheads averaged less than 45 min in summer but increased to a mean of 2–5 h in winter. Maximum dive durations were 7–10 h. Immature loggerheads have been found to similarly change their diving behavior during the winter (Hochscheid et al. 2005, 2007). The torpid, mud-covered loggerheads that Carr et al. (1980) encountered in the Port Canaveral Ship Channel off the east coast of Florida during the winter of 1978 could have been exhibiting this behavior.

We documented unusually long dives for four loggerheads during the winter for the first time in the GOM. All these turtles had primary residence areas on the WFS. Dives by these loggerheads during most of the year lasted for 10–30 min. During the winter (from mid-December through March), dive duration increased dramatically and was often greater than 240 min. We documented this behavior in three loggerheads that had a separate overwintering site and in one loggerhead that did not have a separate overwintering site. The latter loggerhead had a primary residence area in relatively deep water (mean depth of 56 m).

Hart et al. (2012) noted that there was little overlap in the core-use areas at foraging sites of adult female loggerheads in several studies. They suggested that these turtles established individual foraging territories. However, in considering the home ranges of the turtles documented in this study, we believe the apparent territoriality is likely an artifact of a relatively small sample size. We saw substantial overlap in the primary residence areas (as delineated by the *α*-Hull) of three loggerheads on the WFS. The centroids of these residence areas were all within 10 km of each other. In addition, the secondary residence area of one of our loggerheads was entirely within the primary residence area of another loggerhead and both were present in the same area at the same time. Other studies (Limpus et al. 1992; Marcovaldi et al. 2010; Philips 2011) have also documented overlap of adult female loggerhead foraging areas. Additionally, three of us (Foley, Schroeder, and Hardy) have captured hundreds of adult-size loggerheads (>80 cm carapace length), including dozens of females either tagged or intercepted on a nesting beach and more than 100 adult males within a 20-km^2^ foraging area in Florida Bay (eastern GOM; A. Foley, B. Schroeder, and R. Hardy, unpubl data).

The present study found that the foraging areas of adult female loggerheads from Florida were located in at least four countries (USA, Mexico, the Bahamas, and Cuba). This highlights the importance of international collaboration when formulating conservation management plans that are effective at promoting the recovery of loggerhead populations with rookeries in Florida. One such transnational effort is the Inter-American Convention (IAC) for the Protection and Conservation of Sea Turtles. However, of the four countries in our study that share adult female loggerhead foraging areas, only two (USA and Mexico) are currently signatories on this treaty. One of the actions specifically identified in the loggerhead recovery plan is to encourage non-signatory nations (e.g., the Bahamas and Cuba) to accede to the IAC (National Marine Fisheries Service and US Fish and Wildlife Service 2008). Additionally, activities that are conducted within the boundary of one country, such as those related to the extraction of offshore oil resources, could threaten loggerheads living within the boundaries of other nations.

Findings of the present study also inform efforts aimed at preventing or reducing mortality from anthropogenic sources such as commercial fisheries. Incidental mortality of loggerheads in commercial fisheries has a significant impact on loggerhead populations worldwide (Lewison et al. 2004; Lewison and Crowder 2007; National Marine Fisheries Service and US Fish and Wildlife Service 2008; Finkbeiner et al. 2011). Notably, on the WFS, adult female loggerhead foraging sites coincide with the bottom longline portion of the GOM reef fish fishery. It was estimated that 700–1,000 loggerheads were incidentally captured in this fishery on the WFS from the latter half of 2006 through 2007 (Southeast Fisheries Science Center 2008). Developing successful strategies to minimize this fishery’s impact on loggerheads will require specific information on the characteristics of the foraging sites and on the behavior of the loggerheads at these sites. Loggerheads are also killed incidentally when captured in commercial fishing nets, and mortality from trawl fisheries is of major concern for loggerhead conservation in the Northwest Atlantic and GOM (Finkbeiner et al. 2011). Loggerheads in the northern half of the GOM (and perhaps at similar latitudes in the neritic areas of the Northwest Atlantic) may be particularly susceptible to capture by demersal trawling during the winter when dive duration increases significantly and turtles spend much of their time on the bottom, perhaps in a state of unusually low activity.
